# Exploring the differences between pet and non-pet owners: Implications for human-animal interaction research and policy

**DOI:** 10.1371/journal.pone.0179494

**Published:** 2017-06-23

**Authors:** Jessica Saunders, Layla Parast, Susan H. Babey, Jeremy V. Miles

**Affiliations:** 1 RAND Corporation, Santa Monica, California, United States of America; 2 UCLA Center for Health Policy Research, Los Angeles, California, United States of America; Public Library of Science, UNITED STATES

## Abstract

There is conflicting evidence about whether living with pets results in better mental and physical health outcomes, with the majority of the empirical research evidence being inconclusive due to methodological limitations. We briefly review the research evidence, including the hypothesized mechanisms through which pet ownership may influence health outcomes. This study examines how pet and non-pet owners differ across a variety of socio-demographic and health measures, which has implications for the proper interpretation of a large number of correlational studies that attempt to draw causal attributions. We use a large, population-based survey from California administered in 2003 (n = 42,044) and find that pet owners and non-pet owners differ across many traits, including gender, age, race/ethnicity, living arrangements, and income. We include a discussion about how the factors associated with the selection into the pet ownership group are related to a range of mental and physical health outcomes. Finally, we provide guidance on how to properly model the effects of pet ownership on health to accurately estimate this relationship in the general population.

## Introduction

Approximately sixty-two percent of the American population live with a pet [1], and it is generally believed that these pets provide mental and physical health benefits to their human companions [2]. There is a modest but growing research literature examining the health impact of human animal interaction, which is largely inconclusive due to contradictory findings and methodological weaknesses [3]. Several studies have found that owning and/or interacting with a pet (mostly a dog) has benefits for the individual, including mental health outcomes such as decreased anxiety, and physical health outcomes such as improved immune response and physical activity [4–8]. Other studies have documented negative effects of pets including dog bites, spreading of disease, and have shown that pet ownership is associated with asthma and other allergies [4–8], and associations with a higher incidence of heart attacks and readmissions in heart attack patients [9]. And still other studies have found no link between pet owners and health outcomes [10].

If pet ownership is demonstrated to provide mental, social, and/or physical health benefits for adults, children, or adolescents at the population level, it could provide a relatively cost-effective way to promote health. While the published scholarly studies do not provide strong support for a link between pet ownership and health, some evidence does points in that direction, and researchers are calling for stronger methodological studies [3]. There are key limitations common to this body of work that prevent causal links between human animal interaction and health outcomes, even when associations are found. Most problematic, these studies use convenience samples that may not be representative of the general population, examine a narrow range of outcome variables, and use cross sectional designs that do not consider long-term health outcomes. This is not surprising, as experimental designs where people or families are randomly assigned to be pet- or non-pet owners, would be extremely challenging.

Our goals for this paper are twofold: (1) Describe how pet owners and non-pet owners differ. (2) Describe why this difference needs to be accounted for in observational research on pet ownership and health. In this paper, we will examine the factors associated with pet ownership to provide empirical evidence about how dog and cat owners differ from the general population. We also describe how these differences are also associated with health outcomes, which may lead researchers to under- or over-estimate the impact of pet ownership on health in any observational studies that do not use suitable statistical controls. We then provide guidance into how to strengthen the research basis, recommending some recent methodological innovations that help overcome the limitations associated with selection bias.

### Unclear evidence of the relationship between pet ownership and health mechanisms of the potential effect

When examining the relationship between pet ownership and health, it is helpful to first consider the mechanisms through which we believe the effect might work. For example, do pets promote health through companionship and emotional support; do they encourage healthy behavior; or is there something else about them that could improve mental or physical health? Understanding these mechanisms is vital for understanding how pets might impact health so that we can translate findings into broader public health policy.

One research line has focused on how pet ownership may improve the physical health of owners. The leading theory is that pets encourage physical activity [11]. Most dog owners exercise their dogs, and although not the primary aim, exercising one’s dog also usually involves exercising oneself. In an Australian sample the majority of dog owners walked their dog with almost a quarter of them walking together five or more times per week, however dog owners were significantly more likely to exercise at least 90 minutes per week [12]. This benefit may extend to children as well; research found that the odds of being overweight were lower for any young children who lived in a dog owning household [13].

Researchers have also hypothesized the mechanisms through which pets affect psychological wellbeing. Two theories exist regarding the effects of social support—the ‘main effect’ hypothesis suggests that the beneficial effects are diffuse, the ‘buffering’ hypothesis suggests that social support effects are notable only in the presence of stressors. Two experiments provide evidence of these mechanisms, but how these results translate into long term differences in animal owners’ health is unknown. In a test of the buffering effect hypothesis, researchers tested whether animals could moderate the anxiety inducing effects of a stressful situation [14]. The researchers randomly assigned participants to one of five groups: they were asked to pet a rabbit, a turtle, a toy rabbit, a toy turtle, or they were assigned to a control group. The results showed that petting a toy animal was not significantly better than petting no animal at reducing anxiety; however, petting a real animal did significantly reduce anxiety. It should be noted that the sample size was small (58 individuals), focused on a limited set of proximal outcomes, which may not translate into public health outcomes. However, this study suggests that interaction with, and feedback from, the animal may be important in emotional regulation, and the authors hypothesized that the interaction provided a form of social support. Allen, Blascovich, and Mendes [15] tested the buffering hypothesis by subjecting individuals to stressful situations, examining the effects of social support from pet cats, dogs, spouses and friends. The results showed that individuals who had a pet had lower heart rate and blood pressure at rest than those without pets.

Both studies provide strong evidence for stress buffering effects, but their methods limit the researchers’ abilities in concluding that these effects lead to any long term psychological of physical health benefits in a sample drawn from the general population. There is clear evidence of short-term differences in psychological adjustment that may be attributed to animal interactions; however, how these changes translate into public health outcomes for pet owners or within the general population has yet to be determined. Additionally, each study looked at a small range of dependent measures, thus limiting their ability to detect costs or benefits beyond the scope of the study and thus not providing any measure of “net effects.”

Recent work by Beetz and colleagues [16] pulled together the evidence on the impact of human-animal interaction research to present a unified theory on the causal mechanism for the wide array of consistent impacts (e.g., social attention and behavior, interpersonal interactions, mood, heart rate, blood pressure, fear and anxiety, mental and physical health and cardiovascular function) and inconsistent effects (on stress and epinephrine/norepinephrine, immune system functioning, pain management, aggression, empathy, learning). They hypothesize that interacting with animals releases oxytocin, a hormone that is associated with a variety of heath promoting effects, and that the intensity, duration, and type of interaction mediates the relationship between interaction and health outcomes. They support their theory using the findings from 69 empirical studies that ranged in population, methods, measures, and design; however, the theory has yet to be specifically tested.

### Empirical evidence of the effect

The best evidence of the positive effect of animals on physical, mental, and emotional health has focused on a therapeutic environment, termed animal assisted therapy, because the studies use experimental designs that do not suffer from the problems inherent in observational studies [17, 18]. Such research demonstrating the benefits of animals with clinical populations has been carried out primarily with dogs [19], but has also examined the impact of cats [20], horses [21], dolphins [10], guinea pigs [22], and the robotic dog Aibo [23]. The mental health benefits of interacting with animals outside the therapeutic environment have been studied less—in part because of the difficulties of carrying out methodologically rigorous research outside the controlled environment of therapy. The extent to which these studies of the therapeutic environment can be generalized to the presence of pets in the home, and of public health outcomes is somewhat dubious.

The health benefits of human-animal interaction has been also been studied in clinical patients and the findings are contradictory. For example, with adults recovering from illness, some studies have found pet owners do better while others have found that they do worse. Friedmann and colleagues [24] investigated patients who had been discharged from hospital after a heart attack. Patients who owned a dog had a much higher rate of one year survival– 6% of dog owning patients did not survive their first year, compared to 28% of non-dog owning patients. In a follow up study, they further explored this result, finding that there were differences in heart rate variability between pet owners and non-owners who had survived a heart attack, and suggested that this may be a mediating factor in the effect of pets on survival [25]. However, another study found that heart attack patients with dogs were more likely to have another attack or hospital readmission than dog owners [9]. These results demonstrate the potential health outcome differences between pet- and non-pet owners, but since they were performed on small, distinct, and self-selecting populations, they cannot be applied to the general population and we cannot infer that the difference in survival was caused by the dog ownership.

Using non clinical populations, there is more compelling evidence that pet owners may be healthier. One study found that they make fewer visits to the doctor and take less medication [26]. In one of the most methodologically sophisticated studies examining this phenomenon, Headey and Grabka [27] employed propensity score matching to ensure, as far as possible, equivalence in owners and non-owners in Germany. This study represents the most rigorous causal test of pet ownership on overall health, using doctor visits as a proxy. The effect size of the association between pet ownership and doctor visits was reduced after matching across 11 variables—the mean difference between the groups was reduced from .44 visits to .28 visits after selection bias was taken into account. This demonstrates that when the impact of pet ownership is isolated from other related variables that also impact health, the relationship was appears to be diminished (although the authors do not specifically test whether the difference in effect sizes is statistically significant). Overall, they estimated the treatment effect of pet ownership resulted in a 24% reduction in annual doctor visits. They also conducted analyses on an Australia population and estimated an 11% reduction in doctor visits, after controlling for several other demographics that also impact health. While this study clearly demonstrates that isolating pet ownership from confounds is important for precise estimates of its causal impact, it falls short of a definitive answer since there are other differences limiting its generalizability, such as cultural differences between German and Australia samples, the use of doctor’s visits as a proxy for health, potential missing variable bias, among others.

The recent review of the research literature on the impact of pet ownership on health concluded that there is not enough evidence to make any conclusions [3]. The piece reviews the methodological challenges preventing the extant literature from building a strong research base, including problems with small samples, convenience samples, lack of methodological rigor, and self-report measures, and the “file drawer” effect. All the research to date suffers from several limitations that prevent any strong conclusions about the health effects of pet ownership from being made.

### Are pet owners different?

Most of the research on pet ownership and health outcomes compares pet owners with non-pet owners, but is this an appropriate comparison to make? Is there something about pet owners that is inherently different about these groups that may also affect health? In other words, can we trust research that examines pet owners and non-pet owners and then tries to make causal attributions about differences in health? According to some research, pet owners are indeed different across a wide range of variables that are also related to health; however there are only a few empirical studies that help us understand how they may be different and how large that difference may be.

A few studies demonstrate that pet owners are different than non-pet owners in ways that may be important when estimating the impact of pets on any health-related outcomes. For example, dog owners differ from non-dog owners, according to research conducted in Ireland [28]. Apartment and duplex residents were considerably less likely to own dogs (adjusting for household composition and social class), farmers (but only of small farms) were also more likely to own a dog, having children in the house positively predicted dog ownership, and having a cat also predicted dog ownership. The model was less effective at predicting cat ownership—again, apartment dwellers were less likely to own a cat, females were also more likely to own a cat, and there was a slight age effect, with respondents aged from 45 to 64 more likely to own a cat than other age groups. Dog owners were also more likely to also own a cat. In the UK, dog owners tend to be from larger families with females, and young adults and older children, and the presence of other animals such as horses, birds, and cats, were more likely to have a dog [29]. Pet selection effects are rarely accounted for in existing research, making it impossible to separate the potential impacts of pet ownership from the factors that differentiate those who select to live with a pet from those who select not to. In other words, factors that contribute to selecting to have a dog could themselves have health impacts that could be mistakenly attributed to dog ownership.

## Methods

### Data source

This study used survey response data from the 2003 California Health Interview Survey (CHIS 2003), a population-based, random-digit dial (RDD) telephone survey of California households. CHIS is the largest state-level health survey and is designed to provide population-based estimates for the state of California, California counties, and major ethnic groups. CHIS collected extensive information on health status, health conditions, health-related behaviors, health insurance coverage and access to health care services as well as demographic and socioeconomic information. Within each household, separate interviews were conducted with a randomly selected adult (age 18 and over), adolescents (ages 12–17), and parents of children (ages 0 to 11). CHIS 2003 was conducted between August 2003 and February 2004. Interviews were conducted in English, Spanish, Chinese, Vietnamese, and Korean. The demographic characteristics of the CHIS sample (such as race, ethnicity, and income) are very similar to those obtained from Census data, and additional research suggests that CHIS data are representative of the California population [30, 31]. Detailed information about the CHIS methodology is available elsewhere [32, 33], the survey is available online at http://healthpolicy.ucla.edu/chis/design/Documents/CHIS2003_adult_q.pdf, and the data used for these analysis can be accessed at http://healthpolicy.ucla.edu/chis/data/Pages/GetCHISData.aspx under “Public Use Data Files” with the data file name“CHIS 2003 Adult”. All analyses presented in this paper are weighted using the CHIS survey weights which appropriately account for the sample design, nonresponse, and representativeness. The RAND Human Subjects Protection Board reviewed and approved the research for the use of secondary data without any personal identifiers.

### Sample

Our final sample included 42,044 adults for whom Individual characteristics and self-reported cat and dog ownership were available. Of these, 26.2% of respondents owned a dog, 21.5% owned a cat, and 8.5% owned both a dog and cat (these categories overlap). Forty-nine percent of respondents were male; 26.0% were Hispanic, 51.6% were White, 11.7% were Asian, 6.3% were Black, 4.4% were another race; 61.9% were married; and the average respondent age was 44.4. The average household size was 3.3 with a minimum household size of 1 and maximum of 18, 55.9% of respondents owned a home, 66% lived in a house, 56.6% worked full time and 32.2% of respondents had a full-time employed spouse. Among working adults, the average number of hours worked per week was 25.6 and everyone living in the household worked full time in 40.2% of households. Only 7.3% had current asthma, the average BMI was 26.6, and the average self-reported general health was 3.5 (where 1 = poor and 5 = excellent).

### Statistical analysis

We begin by looking at simple weighted differences between pet owners and non-pet owners, and then move on to survey-weighted logistic regression analyses to investigate individual characteristics associated with dog ownership, cat ownership, dog or cat ownership, and dog and cat ownership. This second set of analyses tells us how much a certain characteristic is related to the different types of pet ownership, using log odds to describe the size of the relationship.

We follow this up with two sets of two larger survey-weighted multivariate logistic regression models to see how the regression coefficients change when we include and exclude health variables. Given the cross-sectional nature of this sample, it is impossible to make causal interpretations from any observed associations. We do know that pet ownership cannot change some biological variables, such as gender, age, and race; but it is plausible that pet ownership may influence other variables, including health-related characteristics. Therefore, we model two different regressions: one set that includes variables that can never (or are unlikely to be) changed by pet ownership; and another that includes a set of variables that *might be* influenced by pet ownership. Because our study is cross-sectional, we need to be very careful and precise when interpreting results concerning health-related characteristics.

The regression results are reported using odds ratios, which are transformations of the coefficients from the logistic regressions. The odds ratio for a dichotomous variable should be interpreted as follows: A number over 1 means that this characteristic is positively related to pet ownership, e.g., Home owners OR = 2.72, meaning home owners are 2.72 times more likely to own a dog. A number lower than 1 means that this characteristic is related to a lower odds of pet ownership, e.g., Hispanic OR = .37, meaning Hispanics are 63% less likely to own a dog compared all other race/ethnicity categories. The interpretation of a odds ratio for a continuous-level independent variable is less straightforward because there is no natural baseline, but the direction of the relationship remains the same—an odds ratio above 1 means that the odds of ownership increase as the continuous independent variable increases, and an odds ratio less than 1 means the odds of ownership decrease as the independent variables increases.

In sum, we conducted four types of analyses: (1) a descriptive comparison of the weighted sample characteristics of each group without adjusting for other variables, (2) survey-weighted univariate logistic regression models regressing each type of pet ownership on each of the covariates of interest in its own separate model; (3) survey-weighted multivariate logistic regression models including only socio-demographic variables (models control for all independent variables simultaneously); and (4) multivariate logistic regression models including both socio-demographic and other health variables (models control for all independent variables simultaneously). Each of the multivariate analyses was subjected to a large number of sensitivity and robustness tests, including additional variables and alternative ways of coding age, gender, marital status, and household employment.

## Results

### Pet owners and non-pet owners

Approximately half the sample lived with a cat and/or a dog (48%) and Table 1 provides descriptive statistics for each of our four samples: (1) non-pet owners, (2) dog owners, (3) cat owners, and (4) dog and cat owners. The sample statistics give a picture of how pet and non-pet owners are similar and different. For example, more pet owners are married, female, White, and live in a house. Additionally, the General Health measure is higher; average BMI is lower; but the rates of asthma are higher. We further examine the relationship between ownership and each of these characteristics using survey-weighted logistic regression models in the next sections.

**Table 1:** Pet owners vs. Non owners (Unweighted Ns: All pets = 22,990, All cat owner = 9,039, All dog owners = 11,015, both dog and cat owners = 3,575).

	ALL Non-pet Owners	ALL Cat Owners	ALL Dog Owners	BOTH Dog and Cat Owners
*Variable*	*Weighted % or Mean*	*Weighted % or Mean*	*Weighted % or Mean*	*Weighted % or Mean*
% Male	50.22%	45.69%	47.53%	44.79%
% Female	49.78%	54.31%	52.37%	55.21%
Age (average)	44.08	44.64	44.94	44.36
% Hispanic	34.34%	9.91%	13.87%	7.59%
% White	37.91%	78.60%	71.59%	83.58%
% Asian	15.40%	4.23%	6.32%	2.66%
% Black	8.16%	2.39%	3.62%	1.45%
% Other	4.19%	4.87%	4.60%	4.72%
Household size (average)	3.41	3.04	3.27	3.33
% Home owners	47.69%	64.93%	72.96%	72.60%
% Married	59.80%	63.44%	66.97%	66.43%
Annual household income (average log scale)	10.30	10.77	10.85	10.84
Rural/urban location (average)^1^	1.90	2.17	2.20	2.34
% Live in a house	57.98%	74.55%	83.96%	85.65%
% Everyone in household full time	37.99%	44.33%	43.69%	45.56%
Hours worked per week (average)	24.50	27.69	27.43	28.69
% Employed full time	54.54%	60.69%	59.86%	62.29%
% Spouse employed full time	29.16%	35.85%	38.65%	39.55%
BMI (average)	26.70	26.36	26.52	26.49
% Legally blind	0.89%	1.00%	0.88%	0.98%
% Current asthma	6.39%	9.21%	8.73%	10.04%
General Health^2^ (average)	3.41	3.65	3.64	3.66

^1^ Rural/urban location scale: 1 = urban, 2 = 2nd city, 3 = suburban, and 4 = town and rural

^2^ General health scale: 5 = excellent, 4 = very good, 3 = good, 2 = fair, 1 = poor

### Dog ownership

Table 2 shows the odds ratio of each characteristic being associated with the different categories of pet ownership using survey weights, meaning that we can conclude that when there is a significant difference (p < .05), there is a difference between dog owners and non-dog owners in terms of this characteristic. We find that the following characteristics are associated with higher odds of owning a dog: female, regardless of marital status; married males and females; White; older age; owning a home; better general health; higher household income; more rural location; living in a house; having current asthma; being in a household where everyone works full time; working more hours per week; being full time employed, and; having a spouse that is employed full time.

**Table 2:** Survey weighted univariate regression results–e.g., models that do not control for other variables.

	Dog Ownership		Cat Ownership		Dog and Cat Ownership	
	OR	p	OR	p	OR	P
Male (compared to female)	0.92	<.01	0.84	<.01	0.83	<.01
Married (opposed to non-married)	1.34	<.01	1.09	0.01	1.24	<.01
Male and single (unique effect of being both male and single)	0.76	<.01	0.73	<.01	0.66	<.01
Female and single (unique effect of being both female and single)	0.82	<.01	1.15	<.01	1.02	0.65
Age (10 year increment)	1.02	<.01	1.01	0.22	1.00	0.91
Hispanic (opposed to all other race categories)	0.37	<.01	0.25	<.01	0.21	<.01
White (opposed to all other race categories)	3.14	<.01	4.64	<.01	5.38	<.01
Asian (opposed to all other race categories)	0.43	<.01	0.27	<.01	0.19	<.01
Black (opposed to all other race categories)	0.48	<.01	0.31	<.01	0.2	<.01
Household size (average)	0.99	0.20	0.89	<.01	1.01	0.26
Home ownership (opposed to non-home owners)	2.72	<.01	1.61	<.01	2.23	<.01
Household annual income (log scale)	1.42	<.01	1.26	<.01	1.31	<.01
Rural/urban location^1^	1.35	<.01	1.28	<.01	1.43	<.01
Live in a house (vs. apartment or other)	3.55	<.01	1.67	<.01	3.33	<.01
Hours worked per week	1	<.01	1.01	<.01	1.01	<.01
BMI	1	0.58	0.99	<.01	1	0.57
Current asthma (vs. no asthma)	1.32	<.01	1.4	<.01	1.48	<.01
General Health^2^	1.2	<.01	1.2	<.01	1.18	<.01

^1^ Rural/urban location scale: 1 = urban, 2 = 2nd city, 3 = suburban, and 4 = town and rural

^2^ General health scale: 5 = excellent, 4 = very good, 3 = good, 2 = fair, 1 = poor

Each regression N = 42,044

Given our large sample size, the effect size of these differences must be considered in addition to the significance level. That is, with a large sample size, a difference may be significance but the size of the difference may not be meaningful. Some of our observed differences were quite large and some comparatively small. For example, White respondents were 3.14 times more likely to own a dog, a fairly large difference, while married people are 34% more likely to own a dog, a comparatively smaller difference.

In multivariate models, several respondent characteristics remained associated with dog ownership. Table 3 displays results of the survey-weighted multivariate logistic models including all sociodemographic characteristics, meaning that all the factors were entered into the model simultaneously. These results show that respondents who were female and single, owned a home, lived in a house, had higher annual household income, lived in a more rural location, had a larger household size, and lived in a household where everyone worked full time were more likely to own a dog while respondents of older age and of non-white race were less likely to own a dog. For example, adjusting for all other characteristics, the odds of owning a dog for a respondent who owned a home were 1.56 times the odds for a respondent who did not own a home; the odds of owning a dog for a respondent who *lived* in a house were 2.5 times the odds for a respondent who did not live in house. The differences in dog ownership between the races remained large, with Hispanic, Asian, and Black respondents being 68%, 71%, and 61% less likely to own a dog than White respondents, respectively. These results were robust to multiple ways of entering the variables into the model.

**Table 3:** Survey weighted multivariate logistic regression results predicting pet ownership using model without health-related characteristics–controlling for all variables in the model.

	Dog Ownership		Cat Ownership		Dog and Cat Ownership	
	OR	p	OR	p	OR	p
Male Single^1^	0.913	0.09	0.751	<.01	0.792	<.01
Female Single^1^	1.097	0.03	1.25	<.01	1.297	<.01
Age (10 year increment)	0.907	<.01	0.881	<.01	0.899	<.01
Hispanic^2^	0.321	<.01	0.192	<.01	0.146	<.01
Asian^2^	0.297	<.01	0.177	<.01	0.122	<.01
Black^2^	0.386	<.01	0.194	<.01	0.141	<.01
Other race (not White, Hispanic, Asian or Black) ^2^	0.729	<.01	0.661	<.01	0.63	<.01
Household size	1.037	<.01	0.967	0.03	1.116	<.01
Home ownership	1.562	<.01	1.103	0.04	1.288	<.01
Household annual income	1.068	<.01	1.027	0.07	0.982	0.31
Rural/urban location^3^	1.137	<.01	1.096	<.01	1.198	<.01
Live in a house	2.473	<.01	1.404	<.01	2.356	<.01
Everyone in household employed full time	1.126	<.01	1.147	<.01	1.204	<.01

^1^ Reference category is `married’

^2^ References category is ‘White’

^3^ Rural/urban location scale: 1 = urban, 2 = 2nd city, 3 = suburban, and 4 = town and rural

Each regression n = 42,044

When health-related respondent characteristics were added to the survey-weighted multivariate logistic models (Table 4), all previously observed associations remained, and respondents with a higher BMI and current asthma were more likely to own a dog. For example, the odds of owning a dog for a respondent with current asthma were 1.22 times higher than a respondent without asthma. However, because this is a cross-sectional nature of our study, it is not possible to conclude that dogs cause asthma, that asthma sufferers are more likely to own dogs, or that there is another way that both dog owners and asthma sufferers differ from one another. In addition, while the odds of owning a dog are higher for respondents with a higher BMI, the magnitude of this effect is very small with an OR = 1.006.

**Table 4:** Survey weighted multivariate logistic regression results predicting pet ownership using model with health-related characteristics–controlling for all variables in the model.

	Dog Ownership		Cat Ownership		Dog and Cat Ownership	
	OR	P	OR	p	OR	p
Male Single^1^	0.915	0.10	0.751	<.01	0.789	<.01
Female Single^1^	1.093	0.04	1.241	<.01	1.28	<.01
Age (10 year increment)	0.903	<.01	0.878	<.01	0.890	<.01
Hispanic^2^	0.318	<.01	0.191	<.01	0.142	<.01
Asian^2^	0.302	<.01	0.179	<.01	0.123	<.01
Black^2^	0.379	<.01	0.192	<.01	0.138	<.01
Other race (not White, Hispanic, Asian or Black) ^2^	0.721	<.01	0.655	<.01	0.618	<.01
Household size	1.035	<.01	0.966	0.02	1.112	<.01
Home ownership	1.567	<.01	1.106	0.03	1.3	<.01
Household annual income	1.071	<.01	1.03	0.05	0.99	0.58
Rural/urban location^3^	1.137	<.01	1.095	<.01	1.197	<.01
Live in a house	2.481	<.01	1.408	<.01	2.372	<.01
Everyone in household employed full time	1.126	<.01	1.15	<.01	1.212	<.01
BMI	1.006	0.02	1.003	0.26	1.006	0.132
General health^4^	0.991	0.56	0.991	0.59	0.957	0.07
Current asthma	1.216	<.01	1.191	<.01	1.238	<.01

^1^Reference category is ‘married’

^2^ References category is ‘White’

^3^Rural/urban location scale: 1 = urban, 2 = 2nd city, 3 = suburban, and 4 = town and rural

^4^General health scale: 5 = excellent, 4 = very good, 3 = good, 2 = fair, 1 = poor

Each regression n = 42,044

### Cat ownership

With respect to cat ownership, Table 2 shows similar univariate associations with the odds of owning a cat as seen with dog ownership, with the exception that female single respondents had a *higher* odds of owning a cat (rather than lower), age was not associated with cat ownership, and higher BMI and larger household size were associated with *lower* odds of owning a cat. These differences were all quite small, although the race differences were even more pronounced between cat- and non-cat owners than dog- and non-dog owners: White respondents were 4.64 times more likely to own a cat than respondents from other races.

Multivariate logistic regression results displayed in Table 3 show that single female respondents were 25% more likely to own a cat, while single male respondents were less likely to own a cat when compared to married respondents (reference group) adjusted for all other factors. These multivariate results also show that lower odds of owning a cat are associated with older age and non-white race. Smaller household sizes, home ownership, living in a home, full time employment of the household, and more rural location were associated with higher odds of owning a cat.

When health-related respondent characteristics were added to the model (Table 4), all previously observed associations remained, and respondents with current asthma were 19% more likely to own a cat, after controlling for all the other variables in the model. Again, we cannot make any claims about the direction of the relationship and do not know if cat ownership causes asthma, respondents with asthma were more likely to own cats, or something related to both asthma and cat ownership is behind the relationship. BMI, and general health were not associated with cat ownership after adjusting for other characteristics.

### Dog or cat ownership

Associations between respondent characteristics and dog or cat ownership were similar to those observed for cat ownership alone, so the results are not presented.

### Dog and cat ownership

In univariate analyses, similar relationships to pet ownership were observed when examining dog and cat ownership as compared to dog ownership with the exception that while married status was still associated with a higher odds of pet ownership and single male status was associated with a lower odds of pet ownership, single female status was no longer associated with pet ownership, see Table 1. In multivariate analyses, single male status, older age, and non-white race were associated with lower odds of owning a dog and cat while single female status, larger household size, home ownership, more rural location, living in a house, and full time employment in the household were associated with higher odds of pet ownership. Household income was not associated with ownership in the adjusted model, see Table 3. When health-related respondent characteristics were added to the model, all previously observed associations remained and, similar to results above, respondents with current asthma were more likely to own a dog and cat while BMI, and general health were not associated with pet ownership in the adjusted models, see Table 4.

## Discussion

About one quarter of the sample reported living with a dog, one quarter reported living with a cat, and 8.5% lived with both a dog and a cat. Pet owners differed from non-pet owners across many socio-demographic variables, and these socio-demographic variables either are related to, or can impact, health and other outcomes. Overall, pet owners are more likely to be: single females or married, younger, White, live in more rural areas, live in homes, and belong to households where everyone is employed full time. Additionally, dog owners are more likely to be home owners and have a higher household annual income; and dog and cat owners are more likely to own their own home and have larger households (but there is no relationship to annual household income). In terms of health differences—which should not be considered to be outcomes or predictors of ownership because our study is purely correlational—pet owners were more likely to have asthma, and dog owners were more likely to have higher BMIs; but otherwise, there were no differences between pet and non-pet owners in general health and BMI.

The socio-demographic differences between pet and non-pet owners are not trivial, especially when examining different mental, emotional, and physical health differences across groups—there is a large research literature demonstrating the important role of many of these socio-demographic factors as determinants of health [34–38]. This literature suggests that health varies as a function of a number of sociodemographic factors including age, gender, race, income, education, marital status, employment, and housing. For example, there is a strong inverse relationship between social class and health [39], and it has been estimated that poverty accounts for 6% of mortality in the US [40]. In addition, much research indicates that African Americans and Hispanics have worse health outcomes compared to whites [38, 41].

The research on determinants of health taken together with research examining differences between pet owners and non owners suggests that *some of the health differences observed between pet owners and non owners could be over- or underestimated due to differences in socio-demographic* variables such as age, race, gender, employment, income, and housing, *and not necessarily (or solely) differences in pet ownership patterns*. Indeed, this is exactly what was identified in previous research—once differences in predictors were accounted for, the relationship between pet ownership and doctor visits shrank to half its size [27]. For example, there is ample evidence that socioeconomic status is related to a number of health outcomes [37–39, 41]. The current research found that income and full-time employment were associated with increased likelihood of dog ownership. Therefore, it is possible that some of the positive associations between health and dog ownership found in studies that did not adjust for income could be over- or underestimated due to selection bias.

With this evidence of differences between pet and non-pet owners, studies that attempt to investigate causal relationships between health or other outcomes and pet ownership using observational data should carefully consider methodological adjustments to handle such selection bias. Of the three most common quasi-experimental design choices that seem most applicable to this field of inquiry, we recommend propensity score matching. Propensity score modeling can decrease bias by 58% to 96%, depending on the covariates used in the model and outcome variable [42], but it is by no means the only modeling technique that can help account for potential selection bias in observational data. Other potential methods could use natural experiments and instrumental variable approaches [43], with some potential instruments being different housing policies surrounding pet ownership. Another method, regression discontinuity design [44], may also be employed, particularly if exploring the impact of the dosage of the interaction on health outcomes.

Another potential approach utilizes causal models proposed by Rubin. He proposed a causal model to eliminate group differences on the back end, mimicking the conditions and covariate balance of a randomized controlled trial [45]. His method identifies analytic groups that are precisely matched on all known covariates to identify subsets of similar people and reduce/eliminate the selection bias in analyses. We suggest adjusting for confounding variables using propensity score matching through case weight adjustments. This method models the selection into treatment groups (in this case living with pets) using theoretically important pre-treatment variables and creates case weights that account for the probability that a participant is assigned to the treatment as opposed to the comparison group. This method has been found to be successful at matching groups and obtaining valid treatment effect estimates [42].

Additionally, there has been a great deal of debate about how to select a model to derive the weights. A sophisticated propensity score weighting method using generalized boosted regression, a data-adaptive, nonparametric logistic regression technique was developed at the RAND Corporation which can accommodate complex and nonlinear relationships between covariates and treatment selection [46]. The weights created using boosted regression provide the estimated conditional odds of receiving treatment where *X*_*i*_ is the vector of control variables and *p*(*X*_*i*_) is the estimated conditional probability of receiving treatment for an individual with control variables equal to *X*_*i*_. We believe this approach is most appropriate for causal analyses that model outcomes of pet ownership because the type, strength, and form of the relationship between the predictors and ownership (e.g., “treatment”) have not been empirically established.

While each of these quasi-experimental methods can help reduce selection bias, they also require careful consideration for proper identification of instruments, cutoffs, and covariates. We add another note of caution about the causal direction of the effect of pet ownership on health when using cross-sectional data—deciding whether certain health and/or health-related characteristics should be included in the selection model will be extremely important since the directionality of the cause and effect could go in either direction for some variables (e.g., someone who is not physically active does not adopt a dog vs. someone is less physically active because they do not have a dog).

This research has some limitations. Although we were able to examine the relationship of a large number of socio-demographic variables with pet ownership, there are likely other key selection differences that make the groups nonequivalent. Thus, these findings illustrate that pet and non-pet owners differ, but in no way represent all the differences between the groups. The current research is cross-sectional, and as a result, caution should be taken in interpreting the reported associations. Additionally, California, the state with the largest population in the US, differs from the rest of the country in culture, climate, and geography. Any of these differences may moderate the impact of pet ownership on health which could limit the generalizability to the rest of the country. The data are over a decade old and may not accurately reflect current health trends—however, this study’s main purpose is to demonstrate the importance of selection bias in research, and there is no reason to believe that the presence of selection bias would have changed over time. Finally, one of the largest limitations is that there is no way to determine how long anyone owned a pet, which may be important when examining health outcomes.

## Conclusions and recommendations

Pet owners and non-pet owners differ across many socio-demographic variables, such as gender, age, race, living arrangements, income, and employment status. These differences are also associated with health, so when trying to draw causal inference about pet ownership using a general population sample, selection bias should be accounted for (or at least acknowledged), as it could lead to an over- or under-estimation of pet ownership’s true effects. In our analyses, it appears that it may inflate them, as pet owner characteristics are associated with better mental and physical health outcomes. This is not a new problem, as selection issues have plagued observational research, with many methodologists and statisticians advancing new methods to deal with this problem that used to confound any meaningful analysis. We recommend propensity score matching utilizing boosted regression since the exact relationship between socio-demographic characteristics and pet ownership is unknown.
